# 

**Published:** 1897-03

**Authors:** 


### CONTENTS.

COMMUNICATIONS.

About the Actiou of Arsenious Acid upon the Tooth-Pulp.............. 105

PROCEEDINGS.

Discussion on Cataphoresis .................................................. 108
The Southern Dental Association—Abstract of 1896........................ 129
Mississippi Valley Dental Society............................................. 140

MONTHLY DIGEST.

Bacteriology ............................................................................ 140

SELECTIONS.

Death following the Administration of Nitrous Oxide.................. 146
American Dentists not Wanted in Germany................................ 147
Relation of Alcoholic Indulgence to Insanity.............................. 147
Blood Poisoning after Tooth Extraction...................................... 148
The Prevention of Consumption................................................ 148
The Influence of Exercise on Growth.......................................... 149
Body and Brain Weariness ........................................................ 150
Woman's Sensibility to Pain...................................................... 151
Ex-Medicos.............................................................................. 151

EDITORIAL

The American Medical Association—Dental Section ..................... 152
Western Kentucky Dental Association......................................... 153

OBITUARY.

In Memoriam—Dr. Francis Peabody ............................................ 153
In Memoriam—Dr. James A. Swasey............................................ 155

